# Experimental Study on Millisecond Laser Percussion Drilling of Heat-Resistant Steel

**DOI:** 10.3390/ma18153699

**Published:** 2025-08-06

**Authors:** Liang Wang, Changjian Wu, Yefei Rong, Long Xu, Kaibo Xia

**Affiliations:** 1 Faculty of Mechanical and Materials Engineering, Huaiyin Institute of Technology, Huai’an 223003, China; wangliang@hyit.edu.cn (L.W.); ryfxh96@163.com (Y.R.); 19816090517@163.com (L.X.); 2 School of Mechanical Engineering, Jiangsu University, Zhenjiang 212013, China; xiakaibo@ujs.edu.cn

**Keywords:** millisecond laser, heat-resistant steel, through-hole, blind hole, numerical simulation

## Abstract

Millisecond lasers, with their high processing efficiency and large power, are widely used in manufacturing fields such as aerospace. This study aims to investigate the effects of different processing parameters on the micro-hole processing of 316 heat-resistant steel using millisecond lasers. Through the control variable method, the study examines the impact of pulse energy, pulse count, and pulse width on the quality of micro-holes, including the entrance diameter, exit diameter, and taper. Furthermore, combined with orthogonal experiments and COMSOL Multiphysics 6.2 simulations, the study explores the influence of pulse width on the formation of blind holes. The experimental results show that when the pulse energy is 2.2 J, the taper is minimal (2.2°), while the taper reaches its peak (2.4°) at 2.4 J pulse energy. As the pulse count increases to 55–60 pulses, the exit diameter stabilizes, and the taper decreases to 1.8°. Blind holes begin to form when the pulse width exceeds 1.2 ms. When the pulse width is 1.2 ms, pulse energy is 2.4 J, and pulse count is 50, the entrance diameter of the blind hole reaches its maximum, indicating that longer pulse widths result in more significant energy reflection and thermal accumulation effects. COMSOL simulations reveal that high-energy pulses cause intense melt ejection, while longer pulse widths exacerbate thermal accumulation at the micro-hole entrance, leading to blind hole formation. This study provides important process references for laser processing of through-holes and blind holes in heat-resistant steel.

## 1. Introduction

**Figure 1:**
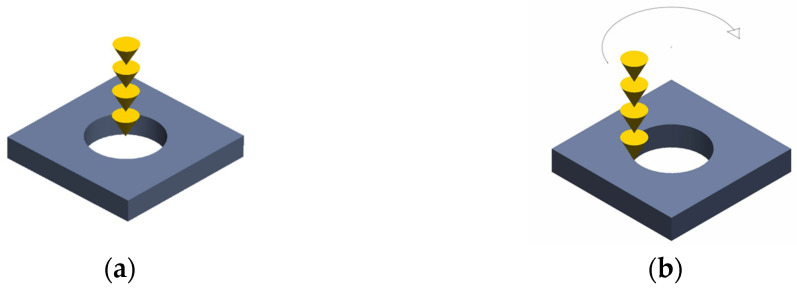
Schematic diagram of laser drilling hole: (**a**) percussion drilling; (**b**) trepan drilling [10].

Heat-resistant stainless steel 316 is a type of austenitic stainless steel characterized by relatively high nickel and molybdenum content, offering excellent corrosion resistance, high-temperature performance, and strong oxidation resistance. These properties make it widely used in aerospace and biomedical fields. For instance, aerospace steels are required to possess high strength-to-weight ratios, thermal stability, corrosion resistance, and fatigue resistance; austenitic stainless steels such as 304, 316, and 321 are commonly applied in engine exhaust systems, fuel systems, and structural components [1]. In the biomedical domain, 316L is extensively used in orthopedic, dental, and cardiovascular implants due to its good workability, corrosion resistance, and biocompatibility [2]. Despite the superior high-temperature mechanical properties, corrosion resistance, and biocompatibility of heat-resistant stainless steels, which make them ideal materials for critical aerospace structures and implantable medical devices, their high strength, hardness, and low thermal conductivity pose significant challenges for conventional machining processes. Traditional methods such as turning, drilling, and milling often lead to rapid tool wear, excessive cutting temperatures, and poor surface finish, particularly in the fabrication of complex microstructures or high-precision holes. These issues significantly hinder their application efficiency and cost-effectiveness in engineering contexts [3,4]. Compared with conventional machining, laser processing exhibits distinct advantages in machining difficult-to-cut materials like heat-resistant stainless steel. Being a non-contact thermal process, laser machining eliminates tool wear, adhesion, and thermal deformation caused by mechanical contact, thereby improving machining stability and tool life. Several studies have identified the non-contact nature of laser processing as a key reason for its high efficiency and reliability. Moreover, laser beams can be focused into micro- to millimeter-scale spots with extremely high energy density, enabling high-precision contour cutting and micro-hole drilling, which is particularly suitable for micro-component fabrication [5,6]. In addition, due to the highly localized heat source of laser processing, the heat-affected zone (HAZ) is significantly minimized, reducing microstructural changes and residual stresses and helping to preserve the mechanical integrity and surface quality of the workpiece. Furthermore, laser parameters such as power, pulse width, and frequency are highly tunable and controllable, allowing flexible adaptation to different material thicknesses and geometries, and extending the application of laser processing to areas such as micro-hole drilling, surface modification, and additive manufacturing [7,8]. Based on processing requirements and material characteristics, laser drilling methods are classified into three categories, as shown in Figure 1: percussion drilling and trepan drilling [9].

As an advanced technique for micro-hole fabrication in heat-resistant steel, laser processing has attracted growing research interest. For example, S. Pattanayak conducted a multi-objective optimization of laser micro-drilling on 316L stainless steel using argon as an assist gas, finding that argon led to thinner recast layers, smaller HAZ, and reduced spatter [11]. H. Chen developed second derivative laser-induced fluorescence (SD-LIF) and intrinsic ratio LIF (IR-LIF) for qualitative and quantitative detection of adulterated edible oils, demonstrating the broader utility of laser-based diagnostics [12]. Zehui Gu investigated the influence of laser angle of incidence (AOI) on picosecond laser micro-drilling, constructing a two-dimensional finite element model incorporating a two-temperature equation and deformable geometry to simulate oblique ablation on stainless steel [13]. Houxiao Wang conducted real-time observation and metallurgical analysis of laser percussion drilling on stainless steel plates assisted by water-based ultrasonic vibration and lateral magnetic fields [14]. Using desirability function analysis, the effects of process parameters on the drilling characteristics of 1.5 mm thick 304 stainless steel were studied, revealing nitrogen pressure as the most influential factor (54.62%), followed by nozzle standoff distance (27.69%) [15]. Hailong Zhang proposed a two-step strategy combining breakthrough and contour modification to fabricate micro-holes on 304 stainless steel, achieving holes with an aspect ratio of 10:1 and taper as low as 0.38° [16]. Wang H. studied the laser drilling process with and without magnetic and/or assist gas, revealing that laser-induced vapor and plasma mix to form a hybrid plume [17]. Houxiao Wang also employed ultrasonic vibration (25 kHz) to assist nanosecond Nd:YAG laser percussion drilling, achieving cleaner, more defined hole walls with reduced defects and taper due to ultrasonic contributions [18]. Tingzhong Zhang implemented a two-dimensional transient model using COMSOL Multiphysics to simulate laser material processing and evaluate spatter dynamics and melt pool behavior [19]. Thus, while extensive studies have been conducted on laser drilling of conventional steels and thin-sheet materials, the drilling mechanism for heat-resistant steels with high melting points and viscosity still requires further investigation. Specifically, the synergistic effects of pulse parameters (energy, width, number) on the morphology of hole entrances/exits and the critical conditions for blind hole formation warrant deeper exploration.

In this study, a DMG CNC precision laser machining center was employed to investigate the laser percussion drilling performance of 316 heat-resistant stainless steel. A combination of the controlled variable method and orthogonal experimental design was used to systematically quantify the effects of millisecond laser parameters—namely pulse energy, number of pulses, and pulse width—on through-hole quality, including entrance diameter, exit diameter, and taper ratio. COMSOL Multiphysics simulations were conducted to further explore the influence of pulse width and pulse energy on hole morphology. The objective of this study is to determine a set of reasonable processing parameters using controlled experiments, and, through the integration of simulation analysis and orthogonal testing, to identify the threshold of laser pulse width required for stable through-hole formation. This work provides both theoretical insight and experimental support for the high-quality application of precision laser drilling in heat-resistant stainless steel.

## 2. Simulation and Experimental Protocol Design

### 2.1. COMSOL Fluid Dynamics Simulation

COMSOL Multiphysics 6.2 supports six geometric configurations: three-dimensional (3D), two-dimensional axisymmetric (2D axisymmetric), two-dimensional (2D), one-dimensional axisymmetric (1D axisymmetric), one-dimensional (1D), and zero-dimensional (0D). Among these, 3D, 2D axisymmetric, and 2D geometries are the most commonly utilized. The 2D axisymmetric option is particularly advantageous for modeling rotational symmetry about a central axis, significantly simplifying the modeling process, reducing computational load, and enabling real-time parameter adjustments.

#### 2.1.1. Modeling and Mesh Generation

**Figure 2:**
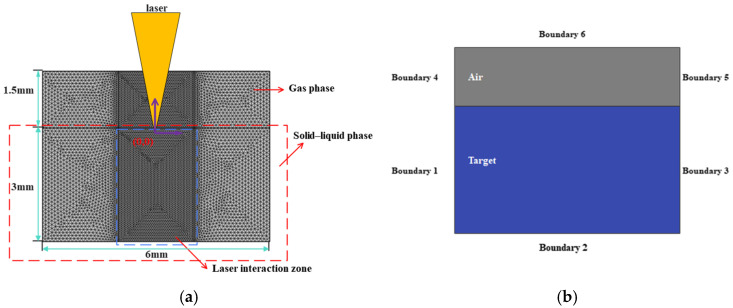
Model mesh generation and boundary conditions, (**a**) model mesh generation; (**b**) boundary conditions.

In this study, a two-dimensional (2D) geometric model is constructed, as shown in Figure 2a, with overall dimensions of 4.5 mm × 6 mm. The material domain measures 3 mm × 6 mm, while the air domain occupies 1.5 mm × 6 mm. A structured meshing strategy is employed throughout the entire model. Following the guideline that the maximum element size should be no greater than one-fifth of the model’s largest dimension, a global maximum element size of 0.1 mm is defined. To improve resolution in the laser–material interaction zone, a refined mesh is applied to a 2 mm-wide central region, reducing the local maximum element size to 0.05 mm.

#### 2.1.2. Heat Source Model

In the experimental setup, the laser beam is focused on the top surface of the workpiece. Correspondingly, in the computational model, the laser energy input is represented by a surface heat source characterized by a spatial Gaussian distribution:

(1) $$I(x)=\frac{2p_{0}}{\pi r_{b}^{2}}\exp\left(-\frac{2x^{2}}{r_{b}^{2}}\right)\delta \left(\Phi \right)$$

In the formula, $p_{0}$ is the laser peak power, and the laser spot radius is set to 0.2 mm. The introduction of the delta function $\delta \left(\Phi \right)$ ensures that the laser pulse energy always acts on the free surface of the small hole.

#### 2.1.3. Initial Conditions and Boundary Conditions

The above simulation process must satisfy the corresponding boundary conditions. The working domain for laser drilling is a two-dimensional plane composed of the metal workpiece and the air above it, as shown in Figure 1b. The initial and boundary conditions are as follows:

1. Initial Conditions

The moment when the laser source starts to act is considered as the initial time, i.e., t = 0.

(2) $$T\left(x,y,o\right)=T_{0}\;,\;u=0,\;P=0$$

2. Boundary Conditions

At boundaries 1, 2, and 3 of the solid metal workpiece:

(3) k∇T=0, u=0

At the left and right outlet boundaries (Boundaries 4 and 5) of the gas region:

(4) $$\left[\mu \left(\nabla u+{\left(\nabla u\right)}^{T}\right)\right]n=0,\;P=P_{0}$$

At the top boundary (Boundary 6) of the gas region, a wetted wall condition is applied:

(5) $$n\cdot u=0,\;\sigma n\delta (\phi )=\frac{\mu }{\beta }u$$

T(x, y, 0) represents the initial temperature field at position (x, y) when time t = 0, T_0_ is the initial uniform temperature, usually corresponding to ambient temperature, u is the fluid velocity vector; setting u = 0 means the material is initially at rest, P is the pressure field, and P = 0 indicates reference pressure (e.g., atmospheric pressure) is assumed, k denotes the thermal conductivity of the solid metal, which characterizes how well heat is conducted through the material, ∇T is the temperature gradient; k∇T = 0 indicates an adiabatic boundary (no heat flux passes through the surface), μ is the dynamic viscosity of the gas, which reflects its internal resistance to flow, σ represents the stress tensor acting on the fluid at the wall, n is the unit normal vector pointing outward from the wall surface, β is the slip coefficient or characteristic slip length.

Considering the complexity of the laser drilling process—which encompasses phase transitions such as melting and vaporization, along with the coupled and dynamic evolution of solid, liquid, and gas phases—the material’s thermophysical properties are assumed to vary both spatially with position and temporally with phase state. Additionally, a mushy zone may exist where the three phases coexist. To simplify the computational modeling, the following assumptions are made:

(1) Chemical reactions between the high-temperature molten material and the surrounding gas are neglected.
(2) The molten liquid within the melt pool is considered an incompressible Newtonian fluid.
(3) The solid/liquid region is treated as an isotropic porous medium.
(4) The effects of plasma formation are ignored.

#### 2.1.4. Material Thermophysical Properties

Since the numerical simulation of the temperature field is nonlinear, it is essential to obtain the thermophysical properties corresponding to different temperatures. The thermophysical properties of heat-resistant steel 316 are shown in Table 1.

### 2.2. Experiment

#### 2.2.1. Experimental Materials

The material employed in this experiment is heat-resistant steel 316 (Baoji Titanium Industry Company, Baoji, China), which maintains stable mechanical and thermal properties at elevated temperatures ranging from 500 °C to 800 °C. It possesses excellent heat resistance, corrosion resistance, plasticity, and oxidation resistance, making it particularly suitable for high-temperature applications and of significant research value in laser drilling studies. The chemical composition of heat-resistant steel 316 is provided in Table 2. The specimen used in the experiment is a semi-cylindrical thin sheet with a thickness of 3 mm and a diameter of 30 mm.

#### 2.2.2. Experimental Equipment

**Figure 3:**
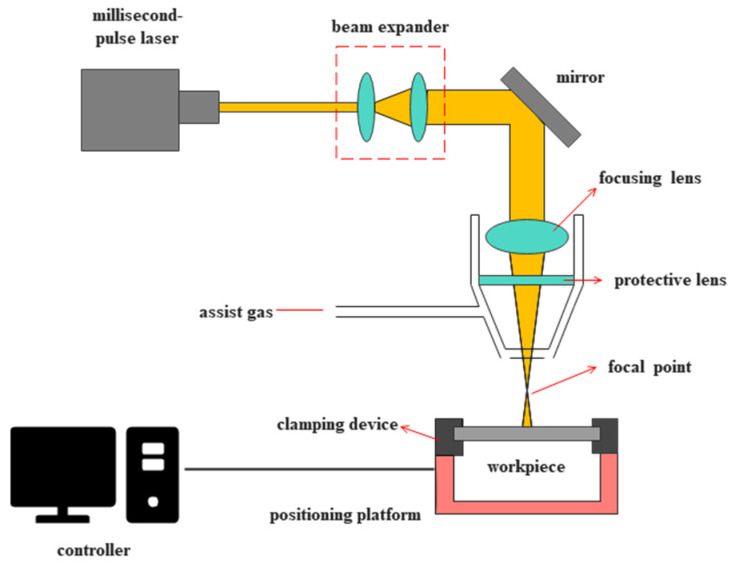
Millisecond laser processing device.

In this experiment, laser processing was performed using the German DMG LASERTEC laser machining center (DMG MORI, Bielefeld, Germany) (Figure 3). The system is equipped with a laser source operating at a wavelength of 1064 nm, with a focus spot diameter ranging from 0.3 mm to 1.0 mm. The pulse width is adjustable from 0.1 ms to 20.0 ms, and the pulse frequency ranges from 0.1 Hz to 500.0 Hz. The system offers a maximum average power of 300 W, a maximum pulse energy of 50 J, and a peak power of up to 20 kW. To improve the drilling quality, nitrogen gas at a pressure of 0.1 MPa was used as the assist gas during the drilling process. The DSX100 optical microscope (Olympus, Tokyo, Japan) was used for morphological characterization. Throughout the experiment, the high-precision capabilities of the laser machining system were utilized to ensure accurate and consistent material processing.

#### 2.2.3. Experimental Method

In this experiment, a control variable approach was employed to systematically investigate the effects of pulse energy, pulse count, and pulse width on micro-hole morphology, including their influence on entrance diameter, exit diameter, and taper. To ensure statistical reliability, each processing parameter was tested at five levels, and three replicate holes were drilled under each condition. This approach minimized random errors and allowed for more robust comparison across different parameter sets. The specific processing parameters are summarized in Table 3.

When investigating the effect of pulse width on micro-hole quality, it was observed that a larger pulse width, under constant pulse energy, leads to a reduction in energy density at the focal point, making it difficult to fully penetrate the material. To minimize the occurrence of excessive blind holes during the evaluation of through-hole quality, the pulse energy was fixed at 2.5 J.

## 3. Experimental Results and Analysis

**Figure 4:**
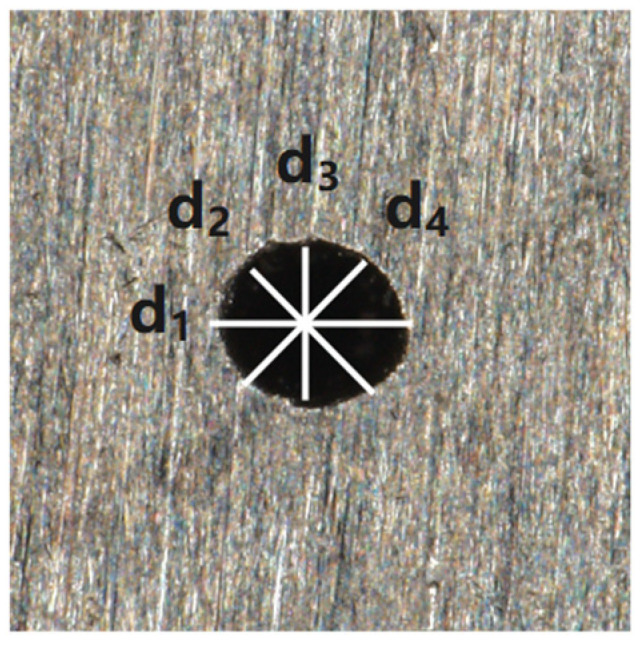
Hole diameter measurement method.

The average value of four diameters was selected with a 45° interval as the hole diameter value, as shown in Figure 4.

The calculation formula for the hole diameter is as follows:

(6) $$d\;=\;\frac{d1\;+\;d2\;+\;d3\;+\;d4}{4}$$

**Figure 5:**
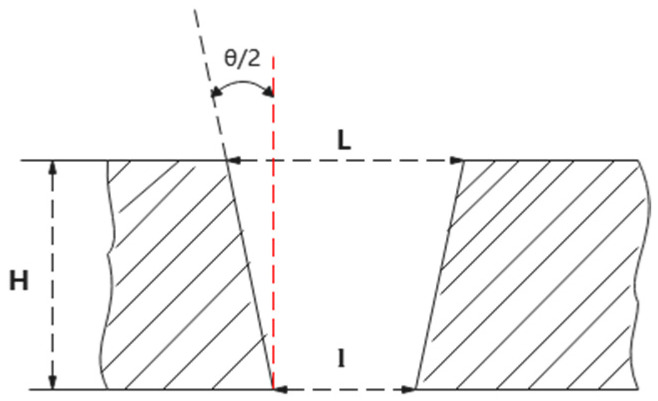
Schematic diagram of the taper in a micro-hole.

The taper is one of the important indicators for measuring the quality of a micro-hole. Figure 5 shows a schematic diagram of the micro-hole taper. The taper θ is defined by the following equation:

(7) $$\theta =\arctan\left(\frac{L-l}{2H}\right)$$

In the formula, “L” represents the entrance diameter, “l” represents the exit diameter, and “H” represents the hole depth (in the case of through-holes).

### 3.1. Through-Hole Research

#### 3.1.1. Influence of Different Pulse Energy on Micro-Hole Quality

**Figure 6:**
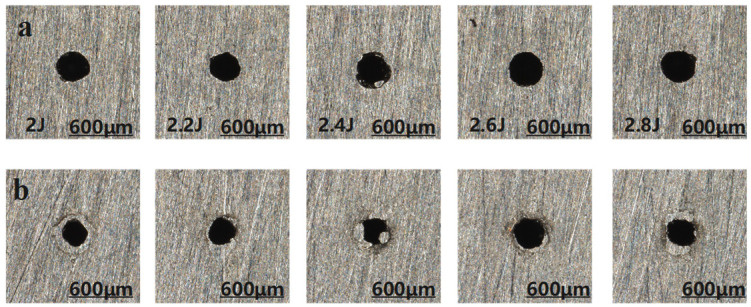
Micro-hole morphologies at entrance and exit under different pulse energies, (**a**) entrance; (**b**) exit.

**Figure 7:**
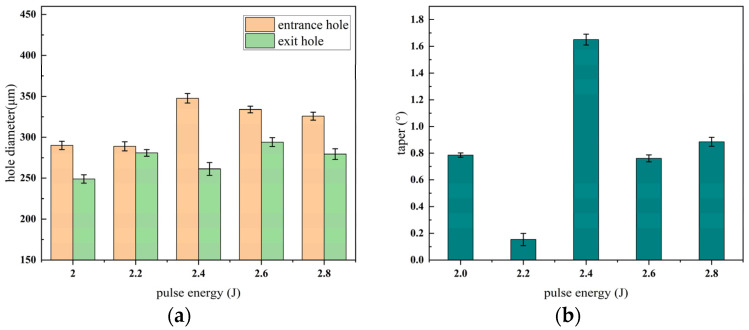
Trend graph of the effect of changing pulse energy on micro-hole diameter and taper, (**a**) hole diameter; (**b**) taper.

The morphology of the micro-hole entrance and exit under different pulse energies during millisecond laser impact drilling is illustrated in Figure 6, where the upper row corresponds to the entrance and the lower row to the exit. The effects of pulse energy on hole diameter and taper are presented in Figure 7.

As shown in Figure 6, the diameters of the micro-hole entrance and exit exhibit significant fluctuations. This behavior can be attributed to the increased energy density of the laser with rising pulse energy under otherwise identical conditions. The higher energy density enhances lateral energy transfer, thereby expanding the effective area of interaction. Consequently, both entrance and exit diameters display notable variations, suggesting that increasing the pulse energy can effectively enlarge the hole diameter during the laser drilling process. According to Figure 7, the entrance diameter initially increases with pulse energy but then gradually decreases, while the exit diameter demonstrates a fluctuating yet overall upward trend. The taper reaches its minimum value at 2.2 J and peaks at 2.4 J. This phenomenon may be due to the fact that, beyond 2.4 J, the entrance diameter becomes less sensitive to the laser spot size, possibly due to saturation effects or the onset of plasma shielding. Additionally, when the pulse energy exceeds 2.2 J, the excessive energy input results in a rapid rise in local temperature, subsequently inducing melting and vaporization of the material and generating a substantial amount of metal vapor. Under sufficiently high laser intensities, this vapor undergoes ionization within the laser field, forming a high-density plasma. Once the plasma reaches a critical density, it acts as a semi-transparent or even opaque medium that absorbs or reflects a considerable portion of the incident laser energy. This significantly hinders the effective transmission of energy to the deeper regions of the workpiece, ultimately leading to a decrease in the exit diameter and an increase in hole taper. Similar observations were reported in the study by Wang H [17]. Therefore, a pulse energy range of 2.2 J to 2.4 J is recommended to balance effective drilling performance and hole quality. According to Tang Q [20], lower energy density leads to more pronounced adverse effects on energy coupling. Conversely, when the laser energy is further increased (2.6–2.8 J), the power density rises, and the local temperature exceeds the absorption threshold of the plasma. As a result, the enhanced ablation capability of the laser surpasses the plasma shielding effect, allowing effective material removal at the hole bottom. This leads to an increase in exit diameter and a reduction in hole taper. However, this improvement comes at the cost of greater laser energy consumption.

#### 3.1.2. Effect of Different Pulse Number on the Quality of Micro-Hole

**Figure 8:**
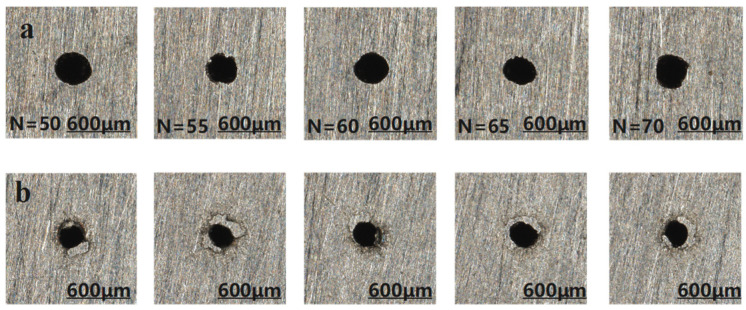
Micro-hole morphologies at entrance and exit under different pulse numbers, (**a**) entrance; (**b**) exit.

**Figure 9:**
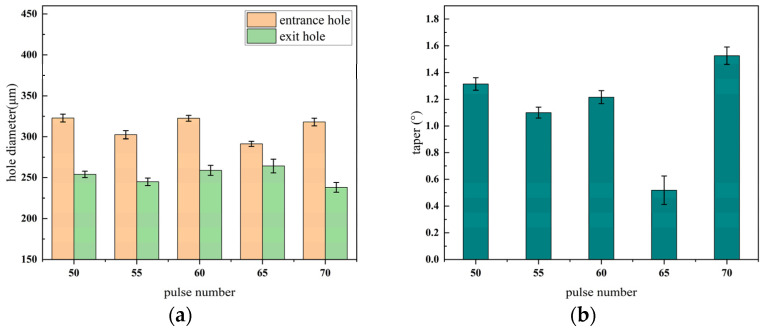
Trend graph of the effect of changing pulse number on micro-hole diameter and taper, (**a**) hole diameter; (**b**) taper.

The entrance and exit morphologies of micro-holes under different pulse numbers during millisecond laser percussive drilling are shown in Figure 8, where the upper row corresponds to the entrances and the lower row to the exits. The influence of pulse number on the aperture diameter and taper of micro-holes is illustrated in Figure 9.

As shown in Figure 8, when the pulse number is relatively low (e.g., 50–55), the entrance and exit of the micro-hole exhibit smooth and well-defined geometries. However, once the pulse number exceeds 60, pronounced ablation appears at the exit, characterized by rough edges, material spattering, and geometric irregularities. This phenomenon primarily results from the prolonged interaction between the laser and the material, which intensifies thermal coupling. The insufficient cooling interval between successive pulses leads to localized heat accumulation—particularly at the hole bottom—causing a continuous rise in temperature. When the local temperature surpasses the boiling point of the material, explosive vaporization may occur. At this stage, the material removal mechanism transitions from melting-dominated to vaporization-dominated, accompanied by micro-explosions and vigorous ejection of material, ultimately resulting in an irregular exit morphology. As shown in Figure 9a, the entrance diameter exhibits minimal fluctuation and reaches saturation early, indicating low sensitivity to increasing pulse number. In contrast, the exit diameter increases steadily up to 65 pulses, reflecting improved laser coupling at the hole bottom and enhanced material removal efficiency. However, at 70 pulses, the exit diameter decreases significantly, primarily due to the onset of plasma shielding. Excessive localized heating leads to the formation of high-density plasma, which absorbs or scatters part of the incident laser energy, thereby impeding effective energy delivery to the bottom region and reducing the material removal rate. Figure 9b further validates this mechanism by illustrating the variation in taper angle with pulse number. Between 50 and 65 pulses, the taper angle gradually decreases, and the micro-hole profile becomes more cylindrical. However, at 70 pulses, a sharp increase in taper is observed due to exit shrinkage, which corresponds to the exit deformation seen in Figure 7. In summary, the entrance diameter is relatively insensitive to pulse number, whereas the exit diameter and taper are highly dependent on it. Excessive pulse numbers induce negative effects such as thermal accumulation and plasma shielding, significantly deteriorating the quality of the micro-hole. Therefore, it is recommended to maintain the pulse number within the range of 55–60 to achieve optimal micro-hole characteristics, including minimal taper, stable morphology, and high processing efficiency.

#### 3.1.3. Effect of Different Width of Pulses on the Quality of Micro-Hole

**Figure 10:**
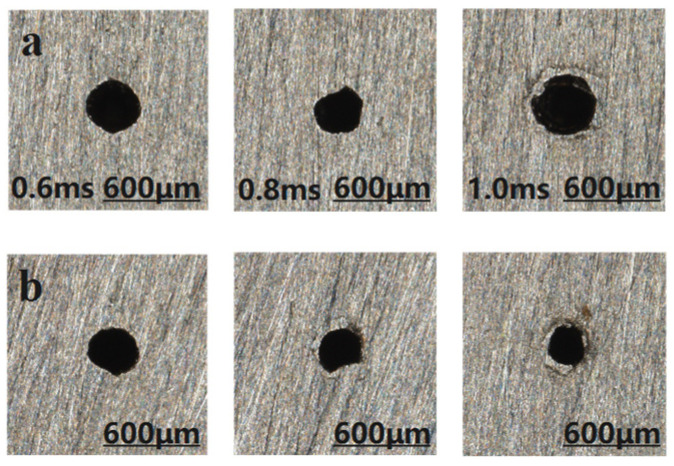
Micro-hole morphologies at entrance and exit under different pulse width, (**a**) entrance; (**b**) exit.

**Figure 11:**
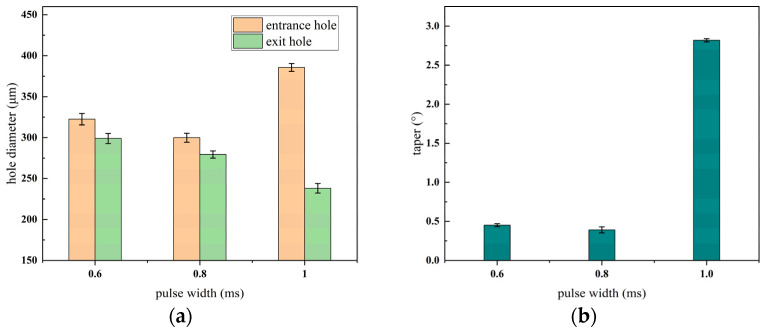
Trend graph of the effect of changing the pulses width on micro-hole diameter and taper, (**a**) hole diameter; (**b**) taper.

The entrance and exit morphologies of the micro-hole under different pulse widths during millisecond laser percussive drilling are presented in Figure 10, where the upper row corresponds to the entrance and the lower row to the exit. The effect of pulse width on the aperture diameter and taper of the micro-hole is illustrated in Figure 11.

As shown clearly in Figure 10, with the continuous increase in pulse width, the accumulation of debris at the entrance of the hole becomes more severe. When the pulse width exceeds 1.2 ms, the micro-hole is not fully drilled through. When the laser repetition frequency and average power remain constant, the pulse energy E, pulse width d and laser peak power $P_{0}$ have the following relationship:

(8) $$E=P_{0}\times d$$

According to the formula, when the pulse energy remains constant, the pulse width and peak power are inversely proportional. As the pulse width increases, the peak power of the laser decreases, resulting in a lower energy density at the focal spot. This reduction in energy density leads to insufficient vapor pressure within the hole, making it difficult to fully eject the molten material. Consequently, some of the expelled debris may adhere to the hole surface under the influence of the assist gas. As shown in Figure 11, with other parameters held constant, the entrance diameter of the hole initially decreases and then increases as the pulse width increases, while the exit diameter exhibits an overall decreasing trend. The taper remains relatively stable at first but gradually increases thereafter. This behavior is attributed to the fact that shorter pulse widths yield higher energy densities, producing larger entrance and exit diameters. However, when the pulse width exceeds 0.8 ms, the cumulative thermal effect of the laser becomes dominant. The energy tends to diffuse both radially and in depth from the point of laser impact, causing the entrance diameter to expand continuously and thus increasing the taper of the micro-hole. Therefore, a recommended pulse width range for optimal processing quality is 0.6–0.8 ms.

### 3.2. Research on Blind Holes

#### 3.2.1. Effect of Different Width of Pulses on the Quality of Blind Hole

**Figure 12:**
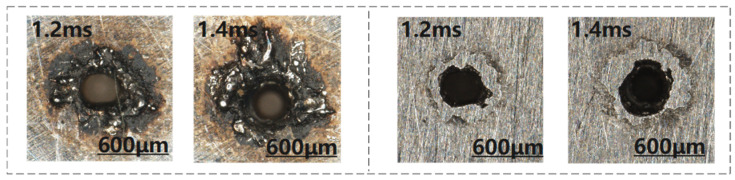
Micro-hole morphology at pulse width of 1.2 ms and 1.4 ms ((**left**): before polishing, (**right**): after polishing).

In the study of the effect of pulse width on the quality of micro-holes, it was found that an excessively large pulse width can produce blind holes. The morphology of the entrance surface of these blind holes is shown in Figure 12.

From Figure 12, it can be observed that at a pulse width of 1.4 ms, debris accumulation at the entrance of the blind hole is significantly greater than that observed at 1.2 ms. This further demonstrates that, with all other factors held constant, a reduction in laser spot energy density diminishes the material’s melting efficiency during drilling. When the pulse width exceeds 1.2 ms, the energy density becomes insufficient to fully melt the material, leading to the formation of blind holes.

#### 3.2.2. The Effect of Other Parameters on the Morphology of Blind Holes

**Figure 13:**
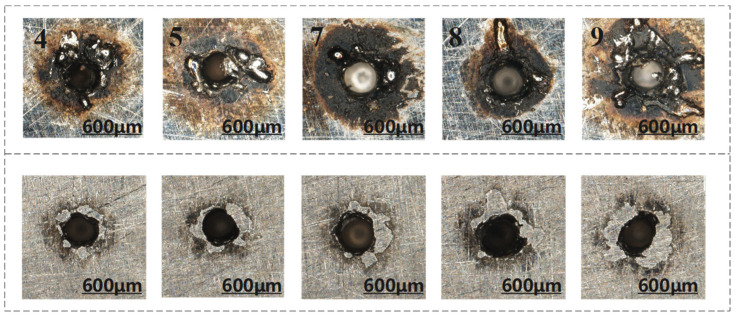
Surface topography of blind hole ((**top**): before polishing; (**bottom**): after polishing).

To explore the effects of other parameters on the formation of blind holes, an orthogonal experimental design was further employed to investigate blind holes in pulsed laser drilling. The data obtained are shown in Table 4, and the resulting morphology of the blind holes is illustrated in Figure 13.

At a pulse width of 1.0 ms, the entrance diameters of the blind holes for sample 4 (2 J) and sample 5 (2.2 J) are 357.7 μm and 376.2 μm, respectively. As the pulse energy increases from 2 J to 2.2 J, the entrance diameter increases significantly, indicating that higher pulse energy enhances material melting and evaporation, thereby increasing material removal at the entrance. Although sample 4 has a higher pulse count (60 pulses) than sample 5 (50 pulses), it exhibits more severe surface ablation. This suggests that, under the same pulse width, a higher number of pulses prolongs the laser–material interaction time, leading to greater thermal accumulation, reduced melt pool stability, and intensified splashing of molten material, ultimately exacerbating surface ablation.

At a pulse width of 1.2 ms, increasing the pulse energy from 2 J to 2.4 J results in an increase in entrance diameter from 395.8 μm to 456.6 μm. Higher energy pulses improve the laser’s penetration capability, enlarging the heat-affected zone near the entrance, which is consistent with observations from through-hole experiments where higher energy correlates with larger entrance diameters. Under larger pulse widths (1.2 ms), both pulse energy and pulse count jointly influence the total heat input. For instance, the entrance diameter of hole 8 (2.2 J, 60 pulses) is significantly larger (453.5 μm) than that of hole 7 (2 J, 55 pulses), indicating that the contribution of energy increase outweighs that of pulse count increase—consistent with the trend observed in through-hole drilling, where pulse energy dominates changes in hole diameter.

The influence of pulse energy on entrance diameter is positively correlated in both blind and through-hole drilling. Higher energy levels lead to more intense molten material ejection and consequently larger hole diameters. In blind hole processing, the effects of energy reflection and thermal accumulation due to incomplete penetration are more pronounced. For example, at a pulse width of 1.2 ms, the entrance diameters of blind holes (395.8 μm to 456.6 μm) are generally larger than those of through holes (302.5 μm to 329.4 μm), indicating that when penetration is incomplete, laser energy concentrates near the entrance, intensifying local melting and expanding the heat-affected zone.

## 4. Numerical Simulation Analysis

### 4.1. Effect of Pulse Energy

**Figure 14:**
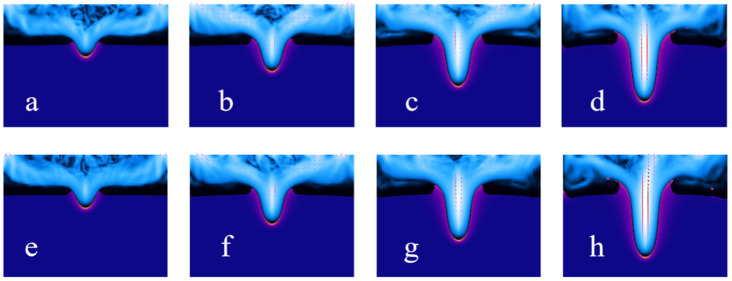
The time-sequence diagrams for the same pulse width under different pulse energies: 2 J (**a**–**d**) and 2.2 J (**e**–**h**), with each time interval being 0.005 s.

Figure 14 is a dynamic sequence diagram of the millisecond laser impact on heat-resistant steel 310 blind hole phenomenon, with a pulse width of 1 ms and pulse energy of 2 J (Figure 14a–d) and a pulse width of 1 ms and pulse energy of 2.2 J (Figure 14e–h).

For the case with a pulse energy of 2 J (Figure 14a–d), the formation of the micro-hole goes through the typical thermal melting and material removal stages. In the initial stage (Figure 14a), laser irradiation causes the local temperature to rise rapidly, melting the material surface and forming the initial melt pool. As the laser continues to act (Figure 14b), the melt pool further expands, and the hole depth gradually increases. In the subsequent stages (Figure 14c,d), the molten region tends to stabilize, the hole shape is essentially fixed, the flow of molten metal weakens, and the boundary of the heat-affected zone (HAZ) gradually becomes clearer. When the pulse energy increases to 2.2 J (Figure 14e–h), the volume of the molten region significantly expands, and the hole formation rate notably increases. Figure 14e,f reflect that the stronger thermal input causes the melt pool to expand more quickly, further raising the local temperature, leading to increased flow of molten metal. By the stages shown in Figure 14g,h, the hole depth significantly increases, the molten region becomes more extensive, and the hole wall is affected by heat to a greater degree. Compared to the 2 J condition, the higher pulse energy promotes the diffusion of molten metal and deepening of the hole, which may also result in a larger thermal accumulation effect.

Overall, the increase in pulse energy significantly enhances the material’s melting and removal effects, causing substantial changes in the depth and shape of the micro-hole. Since the numerical simulation in this study did not consider the plasma effect, the results mainly reflect the thermal impact of laser irradiation on the material and the melting dynamics.

### 4.2. Effect of Pulse Width

**Figure 15:**
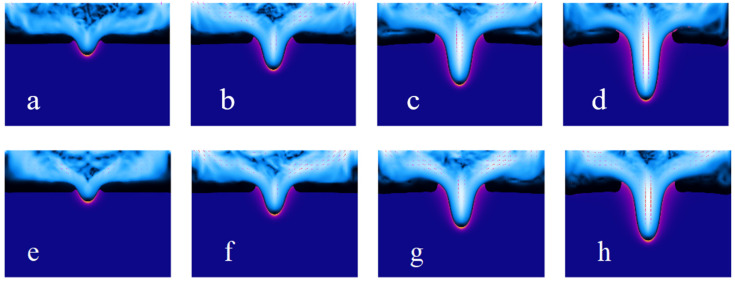
The time-sequence diagrams for the same pulse energy under different pulse width: 1 ms (**a**–**d**) and 1.2 ms (**e**–**h**), with each time interval being 0.005 s.

Figure 15 is a dynamic sequence diagram of the millisecond laser impact on the blind hole phenomenon of heat-resistant steel 310, with a pulse width of 1 ms and pulse energy of 2 J (Figure 15a–d), and a pulse width of 1.2 ms and pulse energy of 2.2 J (Figure 15e–h).

With the pulse energy kept at 2 J, a comparison of the interaction process between the laser and material at pulse widths of 1.0 ms (Figure 15a–d) and 1.2 ms (Figure 15e–h) clearly reveals the influence mechanism of pulse width on micro-hole formation and melting behavior. From Figure 15a–d, it can be observed that at the shorter pulse width (1.0 ms), the laser energy density is higher, and the local heat input is concentrated. The melt pool forms rapidly and extends downward, allowing the micro-hole to reach a significant depth in a shorter time. During this process, the molten region’s shape stabilizes, the heat-affected zone (HAZ) is relatively narrow, and the hole boundary is clear, showing a typical characteristic of deep vertical melting. The higher peak power causes a larger local temperature gradient, resulting in a stronger flow of molten metal and more efficient hole formation. Figure 15e–h shows that when the pulse width increases to 1.2 ms, the energy release process is extended, and the peak power is relatively reduced. The laser heat source presents a more moderate input characteristic. Although the total energy remains the same, the formation speed of the melt pool slows slightly, but its lateral expansion range significantly increases. The molten region becomes broader, the thermal impact on the hole wall becomes stronger, and the heat conduction area expands. The micro-hole’s vertical depth is slightly smaller compared to the shorter pulse, with the molten metal diffusion behavior being more dominant in the lateral direction.

The increase in pulse width significantly affects the energy time-domain distribution and melting dynamics characteristics under the condition of constant pulse energy. Shorter pulses are more conducive to forming deep and narrow hole structures, making them suitable for high aspect ratio machining needs. Longer pulses, on the other hand, enhance lateral heat diffusion, resulting in a broader molten zone and helping to obtain smoother and more transitional micro-hole structures.

The COMSOL simulation in this study was primarily employed to qualitatively investigate the melting behavior and hole morphology evolution during the laser machining process. However, the simulation results were not quantitatively compared with experimental data. As the current simulation focused on qualitative analysis, it cannot directly provide accurate predictions of parameters such as hole diameter or melt depth. To further validate the accuracy of the simulation model, future studies could consider including a verification case, in which simulation results are quantitatively compared with experimental measurements. This would enhance the reliability of the simulation outcomes and provide stronger support for practical applications.

## 5. Conclusions

This paper focuses on the material heat-resistant steel 316, conducting millisecond laser impact drilling experiments to systematically explore the effects of pulse energy, pulse width, and pulse count on the morphology characteristics of through-holes and blind holes. Combining multi-physics field simulations, the study reveals the inherent mechanisms of melting behavior and hole shape evolution. The following conclusions are drawn: In the through-hole processing, pulse energy plays a key role in both hole diameter and taper. The minimum taper (2.2°) is obtained with a pulse energy of 2.2 J, while the taper reaches its peak (2.4°) at 2.4 J. Increasing the pulse width leads to a decrease in exit hole diameter and an increase in taper. Pulse widths exceeding 1.2 ms are likely to result in blind holes. When the pulse count is within the range of 55 to 60, the exit hole diameter tends to stabilize, and the taper is minimized (1.8°). When the pulse width exceeds 1.2 ms, the laser peak power is significantly reduced, leading to insufficient energy input per unit of time, which fails to effectively drive the molten material’s ejection. This results in the deposition of molten material at the hole entrance and the formation of blind holes. This phenomenon is verified through orthogonal experiments and simulation analysis; The simulation results further reveal the mechanism by which laser parameters affect melting behavior. Increasing pulse energy significantly enhances the flowability and splashing ability of the molten metal, accelerating the hole depth evolution. The increase in pulse width leads to energy expansion in the time domain, enhancing the lateral heat diffusion effect, causing the molten zone to widen but limiting vertical development, which is prone to inducing blind hole formation. The research results have significant application prospects in the laser drilling of high-temperature turbine blade cooling holes. By optimizing pulse parameters, the quality and processing efficiency of turbine blade cooling holes can be improved, thereby enhancing engine performance and durability. Additionally, the research results also provide theoretical guidance for micro-hole machining in biomedical devices, such as precision processing of medical instruments.

It should be noted that this study was conducted within a limited range of pulse parameters, and all experiments were performed using heat-resistant steel 316. Although these results provide valuable conclusions, the generalizability of the findings may be limited due to the use of only one material. Therefore, future research should verify whether these conclusions apply to other materials and consider the effects of different laser systems and processing environments. This will help enhance the broader applicability of the conclusions and provide more theoretical support for the application of laser processing technology to other high-temperature materials.

## Figures and Tables

**Table 1:** Thermophysical properties of heat-resistant steel 316.

Physical Properties	Heat-Resistant Steel 310
Solid Specific Heat	500 J/(kg·K)
Liquid Specific Heat	800 J/(kg·K)
Solid Phase Density	7990 kg/m^3^
Liquid Phase Density	7200 kg/m^3^
Solid Phase Dynamic Viscosity	1012 pa·s
Liquid Phase Dynamic Viscosity	0.005 pa·s
Latent Heat of Fusion	2.5 × 10^5^ J/kg
Latent Heat of Vaporization	2 × 10^6^ J/kg
Solid Phase Thermal Conductivity	15 W/m·K
Liquid Phase Thermal Conductivity	10 W/m·K
Thermal Expansion Coefficient	15 × 10^−6^/°C
Solidus Temperature	1673.15 K
Liquidus Temperature	1723.15 K
Melting Temperature	1653.15 K
Vaporization Temperature	3300 K

**Table 2:** Chemical composition of heat-resistant steel 316 (wt.%).

C	Mn	Ni	Si	P	S	Cr	Mo	Fe
≤0.03	≤2	10~14	≤1	≤0.045	≤0.030	16~18	2~3	balance

**Table 3:** Processing parameters for millisecond laser percussion drilling.

Pulse Energy/J	Pulse Width/ms	Number of Pulses
2.0, 2.2, 2.4, 2.6, 2.8	0.8	50
2.5	0.6, 0.8, 1.0, 1.2, 1.4	50
2.0	0.8	50, 55, 60, 65, 70

**Table 4:** Data of orthogonal experimental results (where numbers 4, 5, 7, 8 and 9 are blind holes).

Hole Number	Pulse Width/ms	Pulse Energy/J	Number of Pulses	Entrance Diameter/μm
1	0.8	2	50	302.542
2	0.8	2.2	55	311.630
3	0.8	2.4	60	329.365
4	1	2	60	357.709
5	1	2.2	50	376.210
6	1	2.4	55	346.845
7	1.2	2	55	395.830
8	1.2	2.2	60	453.470
9	1.2	2.4	50	456.587

## Data Availability

The original contributions presented in the study are included in this article; further inquiries can be directed to the corresponding author.
